# Supplementary material for: A diploid assembly-based benchmark for variants in the major histocompatibility complex
Source: Nat Commun. 2020 Sep 22;11:4794. doi: 10.1038/s41467-020-18564-9 (PMC7508831; doi:10.1038/s41467-020-18564-9)
Supplement: Supplementary file 1 — Supplementary Information [file 41467_2020_18564_MOESM1_ESM.pdf]

Supplementary Information for "A Diploid Assembly-based Benchmark for Variants in the Major Histocompatibility Complex" by Chin et al.

Supplementary Note 1: All three technologies were needed to form a single phase block for the MHC

To determine what technologies were necessary to obtain a single phasing block without using trio information, we used WhatsHap 0.18 with various combinations of technologies and calculated the number of phasing blocks:

- PacBio alone: Number of phased blocks: 30, Largest component contains 3095 variants (24.9% of accessible variants) between position 32553266 and 32910482

- PacBio + 10x: Number of phased blocks: 4, Largest component contains 6954 variants (55.9% of accessible variants) between position 28498559 and 31874134

- ONT ultralong alone: Number of phased blocks: 3, Largest component contains 7969 variants (64.1% of accessible variants) between position 28498559 and 32460863

- PacBio + ONT ultralong: Number of phased blocks: 3, Largest component contains 7969 variants (64.1% of accessible variants) between position 28498559 and 32460863 (Note: this results is the same as those of using ONT ultra long alone. We think PacBio reads do not contain extra phasing information on top of the ONT ultra long reads..)

- PacBio + ONT ultralong + 10x: Number of phased blocks: 1, Largest component contains 12441 variants (100.0% of accessible variants) between position 28498559 and 33448264

With our current methods and data types, we need all three data types to achieve a single phasing block across the MHC region.

Supplementary Note 2: Structural Variants (SVs)

Although they are excluded from the benchmark bed, the dipcall vcf also includes 63 deletions and 63 insertions $\geq$50 bp in size. Upon curation of 20 randomly selected SVs, they all appeared to be accurate except for one assembler error in hap1, where the vcf has a false 55 bp deletion at 6:31690555 (near another false 27 bp insertion, both of which are excluded by the benchmark bed). However, 68 out of 126 are within 1000 bp of another SV, and 60 have at least 50 % overlap with a tandem repeat or homopolymer. Clustered SVs like these, particularly in tandem repeats, can typically be represented in many different ways, and unlike small variants, no benchmarking tools currently exist that correctly

compare different representations of clusters of SVs. Therefore, we keep these in the vcf, but future work will be needed to develop tools to use these SVs to evaluate performance in an automated way. One complex example is an inversion and insertion in Supplementary Figure 1A, which is represented by dipcall as a deletion at 6:31009222 and a compound heterozygous insertion at 6:31010095. The haplotype 1 assembly is structurally similar and ~99.6 % identical to an ALT locus in GRCh38 (Supplementary Figure 1B).

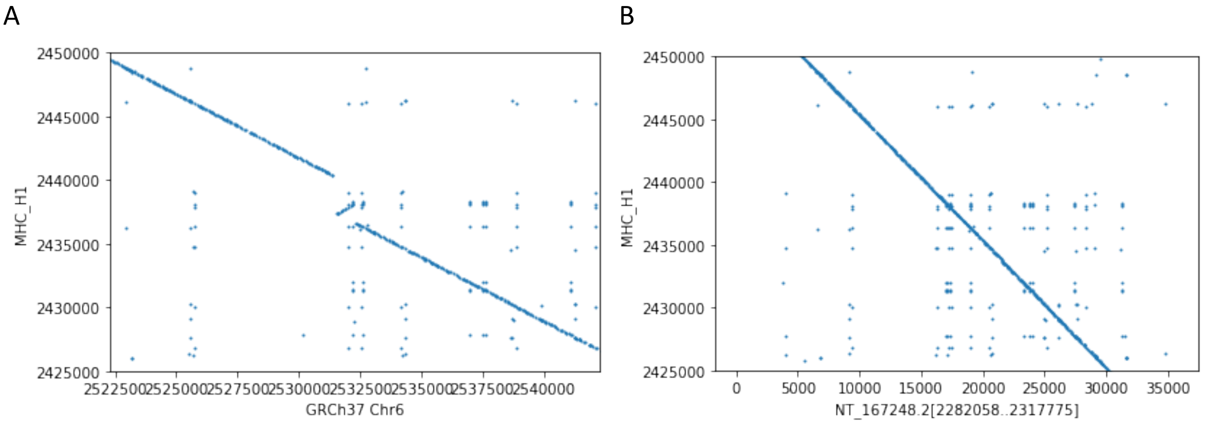

Supplementary Figure 1: A dotplot of the haplotype 1 assembly vs. GRCh37 showing a complex structural variant in HG002, represented as a deletion at 6:31009222 and a compound heterozygous insertion at 6:31010095 in the VCF. The haplotype 1 assembly in this region is structurally more similar to one of the ALT sequence (Genbank accession: NT_167248.2, GRCh38.p12 alternate locus group ALT_REF_LOCI_6 HSCHR6_MHC_QBL_CTG1) in GRCh38. However, the sequence identity between haplotype 1 assembly and the ALT contig is only 99.6%.

Supplementary Note 3: Jupyter Notebooks

We describe the Jupyter notebooks available at https://github.com/NCBI-Hackathons/TheHumanPangenome/tree/master/MHC/e2e_notebooks

00_fetchreads.ipynb: This notebook shows how we fetch reads that may belong to the MHC region of the HG002 Genomes

We use the command shmr-map in the Peregrine Assembler Suite to compare the HiFi reads for phasing and assembling the HG002 MHC region. The 'shmr-map' tools using SHIMMER (https://www.biorxiv.org/content/10.1101/705616v1) to map the reads to the GHRh37 MHC and the (unphased) genome assembly of HG002 MHC region from an assembly (s3://human-pangenomics/HPRC/HG002_Assessment/assemblies/JC_20k_15k_asm/asm.fa.gz, contig 000028F:1700756-6708745). The two MHC sequences are used for recruiting the reads.

01_get_phased_reads.ipynb : Generating Phased Read Sets

This notebook shows the process of performing the haplotype phasing with 10x, Oxford Nanopore Reads and PacBio HiFi reads. We also perform a couple different experiments of different combinations of the data sets for phasing.

02_run_assembler.ipynb: A Notebook for running the Peregrine assembler

This notebook is used in the development for the benchmark assembly. A public docker image registry.hub.docker.com/cschin/peregrine:mhc_hg002_20200325 contains the running environment, data, and the script to reproduce the benchmark assembly.

This notebook only contains the initial assembly part. The assembly contig generated by this notebook does not resolve the repeats around the C4A region. Please see the docker image for the script and code to reproduce our results resolving the repeats.

03_get_variant_cluster: Get variants called from the reads mapped back to the assembled contigs

The assembled contigs should be consistent with the reads that used to generate them. We mapped the reads back to the contigs and checked if there were clusters of variants which indicate potential issues of the assembled contigs.

Reproduce the Phased and repeat resolved assembly with a docker (https://en.wikipedia.org/wiki/Docker_%28software%29) image

Running the docker image in a container and get an interactive shell:

```
docker run -it registry.hub.docker.com/cschin/peregrine:mhc_hg002_20200325
```

Existing assembly results can be found at:

```
/wd/asm-HG002-MHC-H1
```

and

```
/wd/asm-HG002-MHC-H2
```

To regenerate the assemblies in the container:

```
cd /wd && bash generate_assembly.sh
cd /wd/asm-HG002-MHC-H1/ && cp ../template/run.sh . && . /root/.bashrc &&
bash run.sh
cd /wd/asm-HG002-MHC-H2/ && cp ../template/run.sh . && . /root/.bashrc &&
bash run.sh
```

Using Dipcall to call variants in MHC assembly

The following workflow details using Dipcall (commit 7746f3364dab7e9088b059c613fbccc3ff0fc945) to call variants in MHC assembly. We modify the https://github.com/lh3/dipcall/blob/master/run-dipcall#L40 by adding -z200000,10000 to increase the mappability of Minimap2 around the MHC region. The same results can also be achieved by changing -z to 20000,10000.

```
wget -O GCA_000001405.15_GRCh38_no_alt_analysis_set.fa.gz
ftp://ftp.ncbi.nlm.nih.gov/genomes/all/GCA/000/001/405/GCA_000001405.15_GRCh38/seqs_fo
r_alignment_pipelines.ucsc_ids/GCA_000001405.15_GRCh38_no_alt_analysis_set.fna.gz

gunzip GCA_000001405.15_GRCh38_no_alt_analysis_set.fa.gz dipcall.kit/samtools faidx
GCA_000001405.15_GRCh38_no_alt_analysis_set.fa /home/dnanexus/dipcall.kit/run-dipcall
grch38_hg002 /home/dnanexus/GCA_000001405.15_GRCh38_no_alt_analysis_set.fa

/home/dnanexus/hap1_fasta.gz /home/dnanexus/hap2_fasta.gz -x

/home/dnanexus/dipcall.kit/hs38.PAR.bed make -j2 -f grch38_hg002.mak
```

#The command make -j2 -f hg002_denovo_grch38.mak performs a series of operations under the hood.

```
/home/dnanexus/dipcall.kit/minimap2 -c --paf-no-hit -xasm5 -z200000,10000 --cs -r2k -
t8 /home/dnanexus/GCA_000001405.15_GRCh38_no_alt_analysis_set.fa
/home/dnanexus/hap1_fasta.gz 2> grch38_hg002.hap1.paf.gz.log | gzip >
grch38_hg002.hap1.paf.gz

/home/dnanexus/dipcall.kit/minimap2 -c --paf-no-hit -xasm5 -z200000,10000 --cs -r2k -
t8 /home/dnanexus/GCA_000001405.15_GRCh38_no_alt_analysis_set.fa
/home/dnanexus/hap2_fasta.gz 2> grch38_hg002.hap2.paf.gz.log | gzip >
grch38_hg002.hap2.paf.gz

/home/dnanexus/dipcall.kit/minimap2 -a -xasm5 -z200000,10000 --cs -r2k -t8
/home/dnanexus/GCA_000001405.15_GRCh38_no_alt_analysis_set.fa
/home/dnanexus/hap1_fasta.gz 2> grch38_hg002.hap1.sam.gz.log | gzip >
grch38_hg002.hap1.sam.gz

/home/dnanexus/dipcall.kit/minimap2 -a -xasm5 -z200000,10000 --cs -r2k -t8
/home/dnanexus/GCA_000001405.15_GRCh38_no_alt_analysis_set.fa
/home/dnanexus/hap2_fasta.gz 2> grch38_hg002.hap2.sam.gz.log | gzip >
grch38_hg002.hap2.sam.gz
```

```
gzip -dc grch38_hg002.hap1.paf.gz | sort -k6,6 -k8,8n | /home/dnanexus/dipcall.kit/k8
/home/dnanexus/dipcall.kit/paftools.js call - 2> grch38_hg002.hap1.var.gz.vst | gzip >
grch38_hg002.hap1.var.gz

gzip -dc grch38_hg002.hap2.paf.gz | sort -k6,6 -k8,8n | /home/dnanexus/dipcall.kit/k8
/home/dnanexus/dipcall.kit/paftools.js call - 2> grch38_hg002.hap2.var.gz.vst | gzip >
grch38_hg002.hap2.var.gz

/home/dnanexus/dipcall.kit/k8 /home/dnanexus/dipcall.kit/dipcall-aux.js samflt
grch38_hg002.hap1.sam.gz | /home/dnanexus/dipcall.kit/samtools sort -m4G --threads 4 -
o grch38_hg002.hap1.bam -

gzip -dc grch38_hg002.hap1.var.gz | grep ^R | cut -f2- > grch38_hg002.hap1.bed

gzip -dc grch38_hg002.hap2.var.gz | grep ^R | cut -f2- > grch38_hg002.hap2.bed

/home/dnanexus/dipcall.kit/bedtk isec -m grch38_hg002.hap1.bed grch38_hg002.hap2.bed >
grch38_hg002.dip.bed

/home/dnanexus/dipcall.kit/k8 /home/dnanexus/dipcall.kit/dipcall-aux.js samflt
grch38_hg002.hap2.sam.gz | /home/dnanexus/dipcall.kit/samtools sort -m4G --threads 4 -
o grch38_hg002.hap2.bam - /home/dnanexus/dipcall.kit/htsbox pileup -q5 -evcf
/home/dnanexus/GCA_000001405.15_GRCh38_no_alt_analysis_set.fa grch38_hg002.hap1.bam
grch38_hg002.hap2.bam | /home/dnanexus/dipcall.kit/htsbox bgzip >
grch38_hg002.pair.vcf.gz

/home/dnanexus/dipcall.kit/k8 /home/dnanexus/dipcall.kit/dipcall-aux.js vcfpair
grch38_hg002.pair.vcf.gz | /home/dnanexus/dipcall.kit/htsbox bgzip >
grch38_hg002.dip.vcf.gz
```

#For hg19 the command is similar except the reference file were taken from

```
wget -O hs37d5.fa.gz
ftp://ftp.1000genomes.ebi.ac.uk/vol1/ftp/technical/reference/phase2_reference_assembly
_sequence/hs37d5.fa.gz
```

and the PAR file is different dipcall.kit/hs37d5.PAR.bed

HLA*ASM commands

```
perl HLA-ASM.pl --assembly_fasta $assmbly.fa --sampleID $sample --
truthFile truth.txt --use_minimap2 1
```

Commands to generate benchmark regions

https://github.com/NCBI-Hackathons/TheHumanPangenome/blob/master/MHC/benchmark_variant_callset/README_MHC_smallvar_benchmark.txt

	scaffold size	total non-N bases	contig N50	number of gaps	average gap size
GRCh37/38 primary	4,970,557	4,970,557	4,970,557	0	0
GL000250	4,672,374	2,370,831	109,423	28	82,198
GL000251	4,795,265	4,795,265	4,795,265	0	0
GL000252	4,604,811	4,198,717	486,444	10	40,609
GL000253	4,677,643	4,095,121	337,351	16	36,408
GL000254	4,827,813	3,789,326	239,145	22	47,204
GL000255	4,606,388	4,289,729	623,992	5	63,332
GL000256	4,929,269	4,174,253	786,475	17	44,413
H1	4,944,282	4,944,282	4,944,282	0	0
H2	5,036,872	5,036,872	5,036,872	0	0

Supplementary Table 1: Characteristics of contigs in GRCh38 primary and alternate contigs relative to our haplotigs. The primary sequence of the MHC regions is identical in GRCh37 and GRCh38 (except at chr6:28719765 in GRCh38), but GRCh38 has additional ALT loci describing highly divergent sequences, which are in this table.

Contig Coordinates	GRCh37 Coordinates
H1:975575-986243	chr6:32460324-32460749
H1:975575-986243	chr6:32460988-32466089
H1:975575-986243	chr6:32467965-32468122
H1:975575-986243	chr6:32511540-32513868
H2:1073334-1087791	chr6:32108023-32108107
H2:1073334-1087791	chr6:32455269-32458397
H2:1073334-1087791	chr6:32460324-32460749
H2:1073334-1087791	chr6:32460988-32466089
H2:1073334-1087791	chr6:32467965-32468122
H2:1073334-1087791	chr6:32468484-32470163
H2:1073334-1087791	chr6:32472608-32473374
H2:1073334-1087791	chr6:32503470-32503816

Supplementary Table 2: List of low confidence regions in the assembled contigs. After expanding the regions by 10 % of their size and merging, we exclude 6:32452957-32475450 and 6:32501436-32516100 from the GRCh37 benchmark regions.

Parameters	Number of detected variants
-z 200000,10000	26850
-z 200000,1000	26845
-z 200000,200	26845
-z 20000,10000	26850
-z 20000,200	26845
-z 2000,1000	26418
-z 4000,200	26796
-z 2000,200	26796
-z 800,300	23168
-z 400,200	20450
default (-z 200)	18414

Supplementary Table 3: The number of variants from different minimap2 parameters.

Haplotig	Gene	Utilized Ref Contig	start	stop	HG002 Alleles from Clinical HLA Typing	Edit Distance Between Called Genotype vs. Assembly	Closest HLA Type match to haplotig	Haplotype
H1	*HLA-A*	pgf	1436987	1440489	A*01:01:01G	0	A*01:01:01G	Maternal
H1	*HLA-B*	pgf	2848251	2851577	B*35:08:01G	0	B*35:08:01G	Maternal
H1	*HLA-C*	pgf	2763854	2767202	C*04:01:01G	0	C*04:01:01G	Maternal
H1	*HLA-DQA1*	pgf	4086777	4093261	DQA1*01:01:01G	0	DQA1*01:01:01G	Maternal
H1	*HLA-DQB1*	pgf	4110116	4117205	DQB1*05:01:01G	0	DQB1*05:01:01G	Maternal
H1	*HLA-DRB1*	pgf	4029789	4043078	DRB1*10:01:01G	0	DRB1*10:01:01G	Maternal
H2	*HLA-A*	pgf	1437427	1440943	A*26:01:01G	0	A*26:01:01G	Paternal
H2	*HLA-B*	pgf	2843682	2846993	B*38:01:01G	0	B*38:01:01G	Paternal
H2	*HLA-C*	pgf	2768829	2772177	C*12:03:01G	0	C*12:03:01G	Paternal
H2	*HLA-DQA1*	pgf	4182456	4188892	DQA1*03:01:01G	0	DQA1*03:01:01G	Paternal
H2	*HLA-DQB1*	pgf	4201076	4208201	DQB1*03:02:01G	0	DQB1*03:02:01G	Paternal
H2	*HLA-DRB1*	cox	4122938	4138189	DRB1*04:02:01	0	DRB1*04:02:01	Paternal

Supplementary Table 4: Comparison of assembly-based HLA types to trio-phased HLA types from a clinical laboratory. The HLA Type are generated by HLA*ASM
