# Supplementary material for: A diploid assembly-based benchmark for variants in the major histocompatibility complex
Source: Nat Commun. 2020 Sep 22;11:4794. doi: 10.1038/s41467-020-18564-9 (PMC7508831; doi:10.1038/s41467-020-18564-9)
Supplement: Supplementary file 3 — Description of Additional Supplementary Files [file 41467_2020_18564_MOESM3_ESM.pdf]

Description of Additional Supplementary Files

Supplementary Data 1

Description: Detailed results of manual curation of false positives and false negatives from evaluation
