# Supplementary material for: A diploid assembly-based benchmark for variants in the major histocompatibility complex
Source: Nat Commun. 2020 Sep 22;11:4794. doi: 10.1038/s41467-020-18564-9 (PMC7508831; doi:10.1038/s41467-020-18564-9)
Supplement: Supplementary file 5 — Reporting Summary [file 41467_2020_18564_MOESM5_ESM.pdf]

nature research

Corresponding author(s): Justin M Zook

Last updated by author(s): Jul 30, 2020

Reporting Summary

Nature Research wishes to improve the reproducibility of the work that we publish. This form provides structure for consistency and transparency in reporting. For further information on Nature Research policies, see our Editorial Policies and the Editorial Policy Checklist.

Statistics

For all statistical analyses, confirm that the following items are present in the figure legend, table legend, main text, or Methods section.

n/a	Confirmed	
☒	☐	The exact sample size (n) for each experimental group/condition, given as a discrete number and unit of measurement
☒	☐	A statement on whether measurements were taken from distinct samples or whether the same sample was measured repeatedly
☒	☐	The statistical test(s) used AND whether they are one- or two-sided<br>*Only common tests should be described solely by name; describe more complex techniques in the Methods section.*
☒	☐	A description of all covariates tested
☒	☐	A description of any assumptions or corrections, such as tests of normality and adjustment for multiple comparisons
☒	☐	A full description of the statistical parameters including central tendency (e.g. means) or other basic estimates (e.g. regression coefficient) AND variation (e.g. standard deviation) or associated estimates of uncertainty (e.g. confidence intervals)
☒	☐	For null hypothesis testing, the test statistic (e.g. F, t, r) with confidence intervals, effect sizes, degrees of freedom and P value noted<br>*Give P values as exact values whenever suitable.*
☒	☐	For Bayesian analysis, information on the choice of priors and Markov chain Monte Carlo settings
☒	☐	For hierarchical and complex designs, identification of the appropriate level for tests and full reporting of outcomes
☒	☐	Estimates of effect sizes (e.g. Cohen's d, Pearson's r), indicating how they were calculated

Our web collection on statistics for biologists contains articles on many of the points above.

Software and code

Policy information about availability of computer code

Data collection	No software was used
Data analysis	Jupyter notebooks are available for the analyses performed in this manuscript under: https://github.com/NCBI-Hackathons/TheHumanPangenome/tree/master/MHC/e2e_notebooks. These include dipcall v0.1 with GitHub commit 7746f33, minimap2 v2.17, and freebayes v1.3.1-1-g5eb71a3-dirty, The following software used to produce evaluation callsets: GATK v4.0.10.1 HaplotypeCaller, DeepVariant v0.8, Aquila v1.0, Dragen 3.3.7, Variation Graph Toolkit v1.3.1, LongRanger v2.2, and Clair v1.

For manuscripts utilizing custom algorithms or software that are central to the research but not yet described in published literature, software must be made available to editors and reviewers. We strongly encourage code deposition in a community repository (e.g. GitHub). See the Nature Research guidelines for submitting code & software for further information.

Data

Policy information about availability of data

All manuscripts must include a data availability statement. This statement should provide the following information, where applicable:

- Accession codes, unique identifiers, or web links for publicly available datasets
- A list of figures that have associated raw data
- A description of any restrictions on data availability

The assembled haplotigs and benchmark variant calls and regions are available at:
https://github.com/NCBI-Hackathons/TheHumanPangenome/tree/master/MHC/
While other PacBio data were used in the evaluation, described above, the assembly process used PacBio Sequel II System 15kb (2 libraries) and 20kb (2 libraries) CCS/HiFi data18, which are available at:
SRA accessions SRX7083056-SRX7083059 and ftp://ftp-trace.ncbi.nlm.nih.gov/ReferenceSamples/giab/data/AshkenazimTrio/HG002_NA24385_son/

PacBio_CCS_15kb_20kb_chemistry2/
The phasing process used ultralong ONT data, mostly from minion and gridion, which is available at:
SRA accessions SRX7684921-SRX7685027 and ftp://ftp-trace.ncbi.nlm.nih.gov/ReferenceSamples/giab/data/AshkenazimTrio/HG002_NA24385_son/Ultralong_OxfordNanopore/guppy-V3.2.4_2020-01-22/
The phasing process used the 10x Genomics VCF from LongRanger 2.2 (SRA accession SRX2225480), which is available at: ftp://ftp-trace.ncbi.nlm.nih.gov/ReferenceSamples/giab/data/AshkenazimTrio/analysis/10XGenomics_ChromiumGenome_LongRanger2.2_Supernova2.0.1_04122018/GRCh37/NA24385_300G/NA24385.GRCh37.phased_variants.vcf.gz

Field-specific reporting

Please select the one below that is the best fit for your research. If you are not sure, read the appropriate sections before making your selection.

☒ Life sciences ☐ Behavioural & social sciences ☐ Ecological, evolutionary & environmental sciences

For a reference copy of the document with all sections, see nature.com/documents/nr-reporting-summary-flat.pdf

Life sciences study design

All studies must disclose on these points even when the disclosure is negative.

Sample size	This benchmark was created for a single sample because only one sample with all the data needed was available
Data exclusions	No data were excluded
Replication	Replication was not needed here because the goal of the study was to establish a benchmark, and this benchmark was evaluated by 11 independent methods and curators
Randomization	Randomization was not relevant since this was a single sample
Blinding	Blinding was not possible because all Genome in a Bottle data are open and public as soon as possible

Reporting for specific materials, systems and methods

We require information from authors about some types of materials, experimental systems and methods used in many studies. Here, indicate whether each material, system or method listed is relevant to your study. If you are not sure if a list item applies to your research, read the appropriate section before selecting a response.

Materials & experimental systems

n/a	Involved in the study	
☒	☐	Antibodies
☐	☒	Eukaryotic cell lines
☒	☐	Palaeontology and archaeology
☒	☐	Animals and other organisms
☒	☐	Human research participants
☒	☐	Clinical data
☒	☐	Dual use research of concern

Methods

n/a	Involved in the study	
☒	☐	ChIP-seq
☒	☐	Flow cytometry
☒	☐	MRI-based neuroimaging

Eukaryotic cell lines

Policy information about cell lines

Cell line source(s)	GM24385 from Coriell Institute for Medical Research
Authentication	Authenticated by whole genome sequencing
Mycoplasma contamination	All cell lines tested negative for mycoplasma by Coriell prior to aliquotting NIST Reference Material 8391
Commonly misidentified lines (See ICLAC register)	No commonly misidentified cell lines were used in the study.
